# Integrity and Regeneration of Mechanotransduction Machinery Regulate Aminoglycoside Entry and Sensory Cell Death

**DOI:** 10.1371/journal.pone.0054794

**Published:** 2013-01-24

**Authors:** Andrew A. Vu, Garani S. Nadaraja, Markus E. Huth, Lauren Luk, John Kim, Renjie Chai, Anthony J. Ricci, Alan G. Cheng

**Affiliations:** 1 Department of Otolaryngology-Head and Neck Surgery, Stanford University School of Medicine, Stanford, California, United States of America; 2 Department of Molecular and Cellular Physiology, Stanford University School of Medicine, Stanford, California, United States of America; Tokai University, Japan

## Abstract

Sound perception requires functional hair cell mechanotransduction (MET) machinery, including the MET channels and tip-link proteins. Prior work showed that uptake of ototoxic aminoglycosides (AG) into hair cells requires functional MET channels. In this study, we examined whether tip-link proteins, including Cadherin 23 (Cdh23), regulate AG entry into hair cells. Using time-lapse microscopy on cochlear explants, we found rapid uptake of gentamicin-conjugated Texas Red (GTTR) into hair cells from three-day-old *Cdh23^+/+^* and *Cdh23^v2J/+^* mice, but failed to detect GTTR uptake in *Cdh23^v2J/v2J^* hair cells. Pre-treatment of wildtype cochleae with the calcium chelator 1,2-bis(o-aminophenoxy) ethane-N,N,N',N'-tetraacetic acid (BAPTA) to disrupt tip-links also effectively reduced GTTR uptake into hair cells. Both *Cdh23^v2J/v2J^* and BAPTA-treated hair cells were protected from degeneration caused by gentamicin. Six hours after BAPTA treatment, GTTR uptake remained reduced in comparison to controls; by 24 hours, drug uptake was comparable between untreated and BAPTA-treated hair cells, which again became susceptible to cell death induced by gentamicin. Together, these results provide genetic and pharmacologic evidence that tip-links are required for AG uptake and toxicity in hair cells. Because tip-links can spontaneously regenerate, their temporary breakage offers a limited time window when hair cells are protected from AG toxicity.

## Introduction

Aminoglycosides (AG) are potent antimicrobials with devastating side effects of hearing loss. Numerous studies have shown that AGs cause hearing loss by inducing sensory hair cell death through activating reactive oxygen species, lipid peroxidation, and the cell death signaling cascade [1], [2], [3]. Nonetheless, mechanisms of AG uptake into the inner ear are incompletely understood, limiting design of therapeutics aiming at preventing this form of iatrogenic hearing loss. At the cellular level, functional mechanotransduction (MET) channels are required for AG uptake and subsequent toxicity as MET channel blockers prevented these events [4], [5], [6]. Factors increasing the open probability of MET channels enhance drug uptake/toxicity, including hypocalcemia [4], [7], [8], and spatiotemporal maturation of hair cells [6], [9], [10]. Conversely, decreasing MET channel opening via hypercalcemia [7] and myosin7a mutations [11] limits AG entry and subsequent sensory cell loss. More recently, genetic knockout of transmembrane-channel like proteins restricted uptake of the AG gentamicin [12]. However, alternative entry pathways via endocytosis [13] and transient receptor potential channels [14] have been described, raising the possibility that blocking MET channel alone is not sufficient in limiting AG toxicity.

As part of the MET machinery, tip-links span stereocilia in hair cells [15] and regulate MET channel opening [16], [17]. Tip-links consist of Protocadherin 15 (Pcdh15) and Cadherin 23 (Cdh23) [18], [19], [20], [21], [22], with their structural integrity being calcium dependent [16], [23]. Correlating with the loss of tip-links, hair cells treated with calcium chelators have reduced or absent MET channel activity [16], [24], [25], [26], [27]. Like hair cells subjected to calcium chelation, hair cells deficient in Pcdh15 or Cdh23 show fewer tip-link-like structures [17], [20]. Although others have reported tip-links in Cdh23 null mice [28], Pcdh15 and Cdh23 deficient hair cells demonstrate reduced MET channel activity [17], [20], [29]. After recovering from calcium chelation, hair cells spontaneously regain tip-links and mechanosensitivity in a Cdh23-dependent manner [23], [24].

The relationship between tip-link integrity and AG uptake in sensory hair cells has not been thoroughly investigated. In this study, we show that Cdh23 deficiency and calcium chelation disrupting tip-links abolished AG uptake and prevented subsequent sensory hair cell death. In addition, the AG uptake mechanism returns within 24 hours of calcium chelation, correlating with the time course of tip-link regeneration. Thus we conclude that the tip-link complex is required for AG uptake and toxicity and that this is the major pathway of uptake under normal conditions that lead to hair cell degeneration.

## Materials and Methods

### Animals

*Cdh23^v2J^* transgenic mice in C57Bl/6 background (Stock number 002552, Jackson Laboratory, Bar Harbor, ME) were used with genotypes determined by sequencing. Wildtype mouse (C57Bl/6) pups were obtained from Charles River laboratory (Cambridge, MA). All procedures involving these animals were approved by the Stanford University administrative panel on laboratory animal care (Assurance number A3213-01, Protocol ID 18606).

### Organotypic Cochlear Cultures

Cochleae were isolated and cultured as previously described [6], [30]. Briefly, cochleae were isolated under sterile conditions in Hank’s Balanced Salt Solution (HBSS, Cellgro, Manassas, VA) from postnatal (P) three-day-old mice. Stria vascularis and modiolus were removed before organs were attached onto 10 mm glass coverslips pre-coated with CellTak (BD Bioscience, San Jose, CA). Each organ was cultured in Dulbecco’s Modified Eagle Medium (DMEM)/F12 (Invitrogen, Carlsbad, CA) culture media containing 10% fetal bovine serum (FBS)(Omega Scientific, Tarzana, CA) and ampicillin (50 µg/ml; Sigma, St. Louis, MO) in 4-well 35 mm tissue culture dishes (Greiner Bio-one, Monroe, NC) for 1–3 days at 37°C in a 5% CO_2_ atmosphere [31], [32]. Media was refreshed every 24–48 hr.

After an overnight culture period, cochleae were exposed to gentamicin (Gentamicin solution, Hospira, Lake Forrest, IL,) for 1 hr, after that they were washed and incubated in fresh culture media for an additional 48 hr. In separate experiments, gentamicin-conjugated Texas Red (GTTR)(1 µM), which was synthesized from gentamicin sulfate (Sigma) and succinimidyl esters of Texas Red dye (Invitrogen) as previously described [33], was also added for 1 hr after an overnight culture period. Separately, cochlear organs that had been cultured overnight were bathed in FM1-43 (5 µM in HBSS×15 sec)(Invitrogen) then imaged under an Olympus SZX10 microscope (Olympus, Center Valley, PA) with epifluorescent optics and FITC filters (488 nm excitation and 520 nm emission) [34].

For calcium chelation experiments, BAPTA (5 mM in DMEM/F12, containing 1.05 mM calcium chloride (Invitrogen) with 10% FBS and ampicillin) was added to cochleae as a steady stream aiming at the organ, followed by incubation for 15 min at 37°C with gentle stirring every 5 min. As previously shown this BAPTA solution breaks >95% of the tip-links resulting in the loss of MET current responses [16], [23], [35]. Subsequently, organs were incubated in BAPTA-free media for 10 min, 6 hr, or 24 hr before gentamicin or GTTR treatment. We found that adding BAPTA-containing media as a steady stream aiming at the organ generated more consistent results than when it was added as droplets onto the cochleae or steady stream directed away from the cochleae (Figure S1). Therefore, the former method of application was used for all remaining experiments.

### Immunohistochemistry

Procedures have been previously described [30]. Briefly, at the end of defined culture periods, tissues were fixed in 4% paraformaldehyde (in phosphate-buffered saline (PBS), pH 7.4) for 30 min, rinsed with PBS (3X), and immersed in blocking solution (5% normal goat serum, 0.1% Triton X-100, 1% bovine serum albumin, and 0.02% sodium azide in PBS, pH 7.4) for 60 min. Primary antibodies diluted in blocking solution were applied overnight at 4°C. The next day, tissues were rinsed with PBS (3X) and then exposed to fluorescent secondary antibodies diluted in PBS with 0.1% Triton X-100, 1% bovine serum albumin, and 0.02% sodium azide for 60–120 min. After washing in PBS (3X), organs were mounted in fluorescent mounting media (Dako, Carpinteria, CA) and analyzed. The following antibodies were used: anti-myosin7a antibody (1∶1000; Proteus Bioscience, Ramona, CA); anti-gentamicin antibody (1∶200; QED Bioscience, San Diego, CA); and corresponding secondary antibodies (Alexa Fluor 488 and 546; 1∶500; Invitrogen).

### Live Imaging of Gentamicin Uptake Using Two-Photon Microscopy

We followed previously described procedures for live imaging [6]. Briefly, acutely isolated cochleae were secured onto sterile 35×10 mm tissue culture dishes (Greiner Bio-one). Organs were cultured overnight, then washed 3X with L-15 before two-photon imaging. GTTR (3 µM) was directly added to cochlear cultures. An Olympus BX-61 microscope (Olympus) with a 100X, 1.0 numerical aperture water immersion objective (Olympus LUMPlan) was used to image the middle turn of the cochlea. A 520 nm long-pass dichroic (Chroma Technology, Bellows Falls, VT) was used to separate the fluorescence emission into two channels and detected by photomultiplier tubes. Using Prairieview software at 1.4X digital magnification (Prairie Technologies, Middleton, WI), we ran 60 min time series at 1 min intervals. A z-position between the nuclei and apical surface of outer hair cells was selected. Femtosecond pulses of 900 nm illumination from a tunable Chameleon XR laser (Coherent Inc., Santa Clara, CA) were scanned across the sample with an average power of 3±0.5 mW to excite the Texas Red fluorophores. Red fluorescence originating from GTTR was isolated using a custom made bandpass filter (620/60, Chroma Technology). Prior to adding GTTR, we scanned the sample at 740 nm and detected blue NADH intrinsic fluorescence with a custom made bandpass filter (480/30) to confirm the health of the sample [36]. Samples with low NADH signals were discarded.

To quantify the fluorescence intensity, ten individual cells were selected as regions of interest (ROIs) in each T-series. Using Image J software (NIH), the pixels within the ROIs were averaged to obtain a fluorescence intensity measure for each cell. The average background intensity was negligible and therefore was not subtracted from the total fluorescence. The fluorescence for the ten cells was averaged to create a single average fluorescence per T-series. The average of each T-series was normalized to the maximum fluorescence and this normalized average and the standard deviation was plotted as a function of time (OriginLab, Northampton, MA) to determine the average increase in cellular fluorescence over time after GTTR administration.

### Image Analyses and Statistics

**Figure 1:**
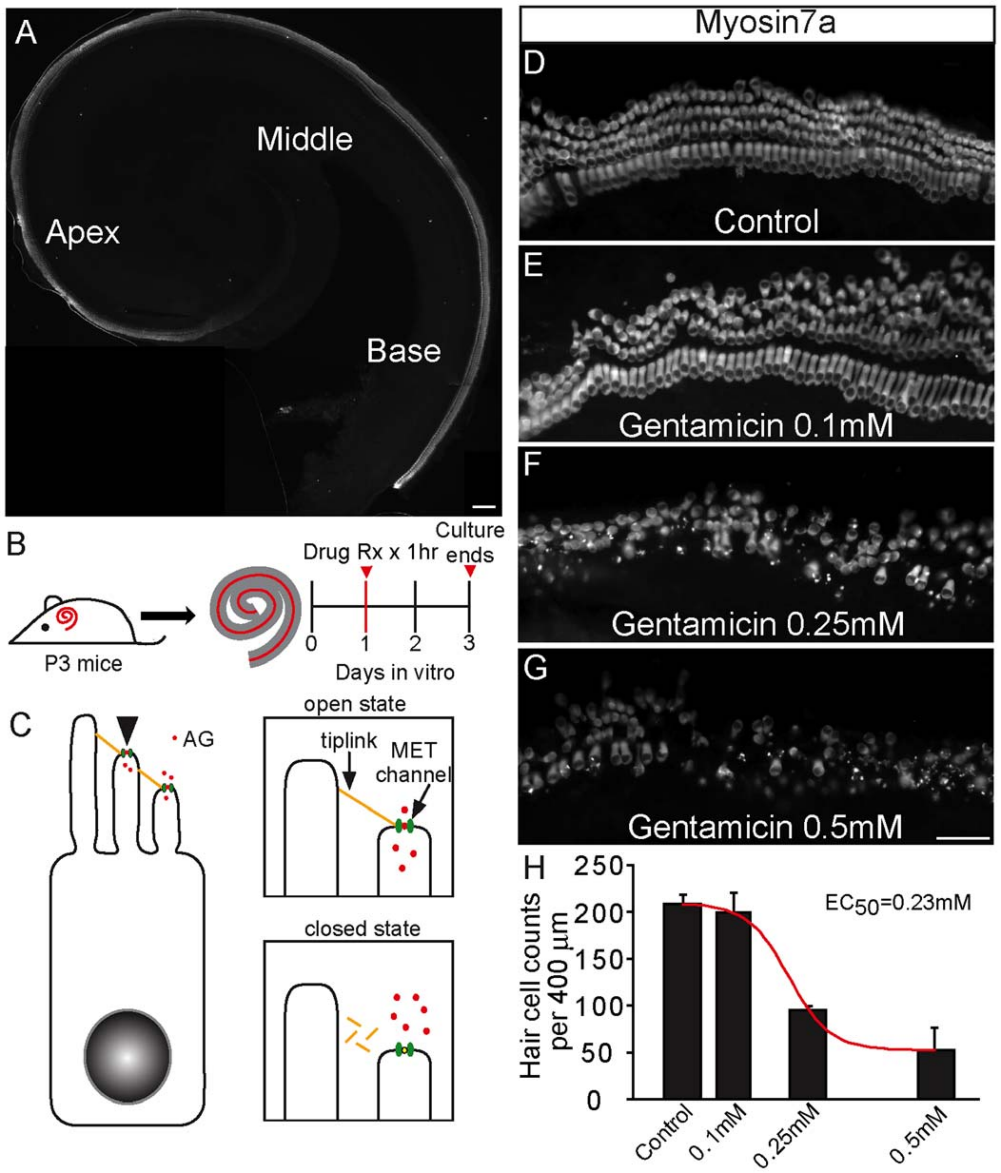
Gentamicin causes cochlear hair cell loss in a dose-dependent manner. A) Low magnification image of a postnatal 3-day-old (P3) mouse cochlea immunolabeled for myosin7a, a specific marker for inner and outer hair cells. B) Cochleae were isolated from P3 mice and cultured overnight. The following day, cultures were bathed in gentamicin (0–0.5 mM) for 1 hr at 37°C followed by a 48 hr recovery period in AG-free culture media. C) Schematic of aminoglycoside (AG, red) entry into hair cells via mechanotransduction channels (MET, green) located at the apical end of stereocilia, and proposed mechanism of MET channel closure preventing AG entry following tip-link (yellow) breakage. D–G) Representative images of the middle turn of cochleae treated with varying doses of gentamicin and labeled with anti-myosin7a. (D) Control. (E) 0.1 mM gentamicin (F) 0.25 mM gentamicin (G) 0.5 mM gentamicin. H) Myosin7a-positive hair cells per 400 µm middle turn were counted and 0.23 mM gentamicin was determined to cause a 50% hair cell loss, mostly among outer hair cells. Error bars = S.D., scale bars = 100 µm in A, 25 µm in D–G.

Tissues were imaged using a Zeiss Axiovert LSM 5 Pascal confocal microscope. The cochlea was divided into apical, middle and basal turns (Figure 1), which were separately analyzed. For hair cell quantification, myosin7a-positive hair cells were counted per cochlear length using the cell counter in Image J software. For the gentamicin dose-response curve and experiments on the *Cdh23^v2J^* mice, hair cells from the middle turn of the cochleae were counted per 400 µm. In experiments testing BAPTA treatment, hair cells from all three turns of the cochleae were counted over a 225 µm length of the organ of Corti.

To quantify GTTR fluorescence in fixed tissues, images were captured from each turn using a Zeiss LSM5 Confocal microscope, with a 40X 1.3NA Plan Neofluor oil immersion objective and identical microscope settings (546 nm laser excitation, emission filter BP 560–615 nm, pinhole at 1 Airy unit, detector gain at 763). The identical settings were used for experimental and control groups from all trials. The cytoplasm of myosin7a-positive hair cells between the level of the cuticular plate and nucleus was selected as the ROI’s. As above, ROI’s were averaged using Image J and intensities of GTTR fluorescence were normalized to the brightest cell in each of the three culture durations. To assess the time course of GTTR uptake, best fit curves were produced using the formula Y = Ae^(−x/t)^, from which the time constants (t_1/2_) were derived. Normalized intensity histograms (10% bin widths) were generated and fit with Gaussian functions to identify intensity peaks and full width half maximums. Either single or double Gaussian fits were used.

Image preparation for figures was performed using Photoshop (Adobe Systems, San Jose, CA) software. Graph preparation and data analyses were done using Microsoft Excel (Redmond, WA) and Origin softwares. Two-tailed Student’s t-test (unpaired) was used for statistical comparison and p<0.05 considered significant.

## Results

### Cadherin 23 Mutation Prevents AG Uptake and Hair Cell Loss

We first established a dose-response relationship between the AG gentamicin and hair cell survival using organotypic cultures of cochleae from postnatal-three-day old (P3) mice (Figure 1A). This *in vitro* system is an established model to study sensory hair cells and AG toxicity [6]. After an overnight incubation period, organs were bathed in gentamicin for 1 hr (0–0.5 mM), followed by a 48-hr AG-free culture period allowing hair cell degeneration to complete (Figure 1B) [6]. Myosin7a is an unconventional myosin expressed in both outer and inner hair cells, and was used to mark surviving hair cells. Increasing doses of gentamicin resulted in an increased hair cell death as measured from the middle turn of cochlear cultures, with 46% myosin7a-positive hair cells remaining after treatment with 0.25 mM gentamicin (Figure 1D–H).

**Figure 2:**
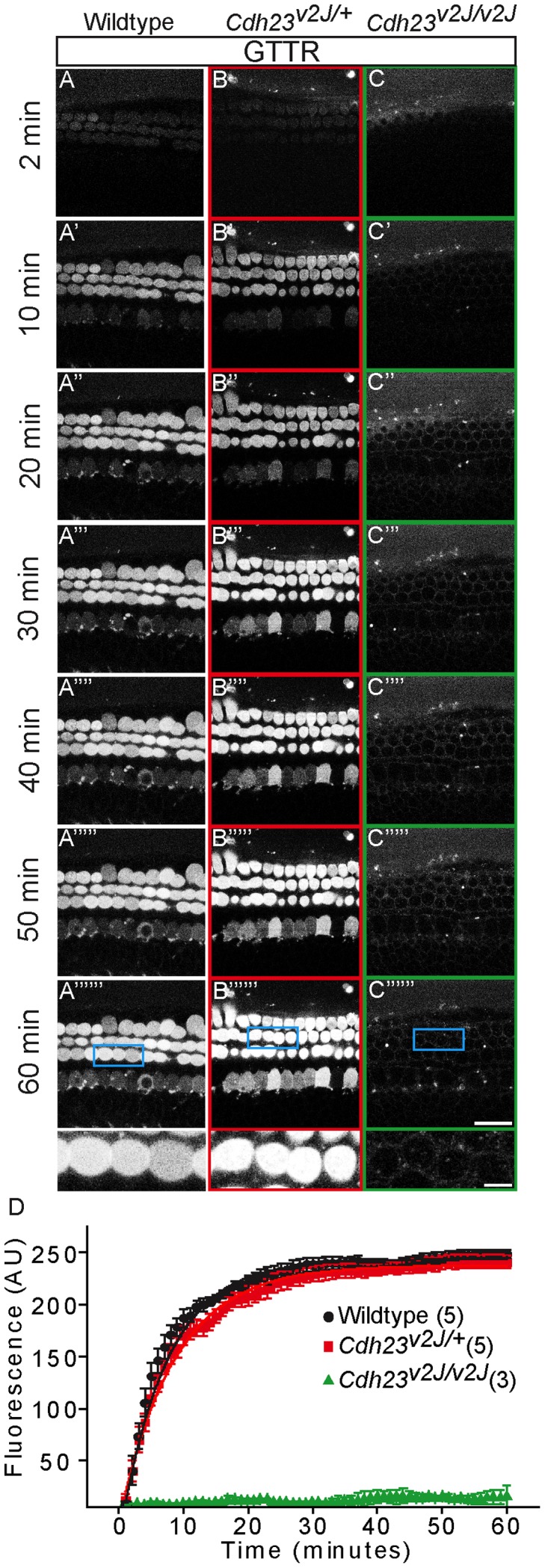
Two-photon time lapse imaging of GTTR uptake into live *Cdh23^v2J^* transgenic mouse cochlear hair cells. P3 cochleae of mouse litters from *Cdh23^v2J/+^* breeding were isolated and cultured overnight before treatment with GTTR (3 µM×1 hr). A–C) GTTR rapidly entered into outer hair cells of wildtype and *Cdh23^v2J^* ^/+^ cochleae, whereas GTTR did not enter hair cells of *Cdh23^v2J^* ^/v2J^ cochleae. Insets depict magnified views of outer hair cells of cochleae of each genotype after exposure to GTTR for 60 min. D) Quantification of fluorescence intensity (AU = arbitrary units) shows that the rate of GTTR uptake among outer hair cells from wildtype and heterozygous mice were comparable, while those from homozygous mice had no detectable drug uptake. Error bars = S.D., scale bar = 20 µm in A–C and 5 µm in insets.

To study the time course of AG uptake, we conjugated gentamicin with Texas Red as previously described [33] and applied it to cultured cochlear organs. To test the hypothesis that tip-links are required for AG uptake into hair cells, we studied the *Cdh23^v2J^* mouse line, which has a point mutation resulting in dysfunctional alternative splice forms and deficient tip-links [17], [37]. Time lapse imaging using two-photon microscopy revealed rapid uptake of gentamicin-conjugated Texas Red (GTTR) into wildtype and *Cdh23^v2J/+^* sensory hair cells (Figure 2A–B), with t_1/2_ being 8.7±0.2 and 7.4±0.2 min, respectively (Figure 2D). By contrast, GTTR was not detected in *Cdh23^v2J/v2J^* hair cells within the same timeframe (t_1/2_ = 2708±513 min)(Figure 2C–D).

**Figure 3:**
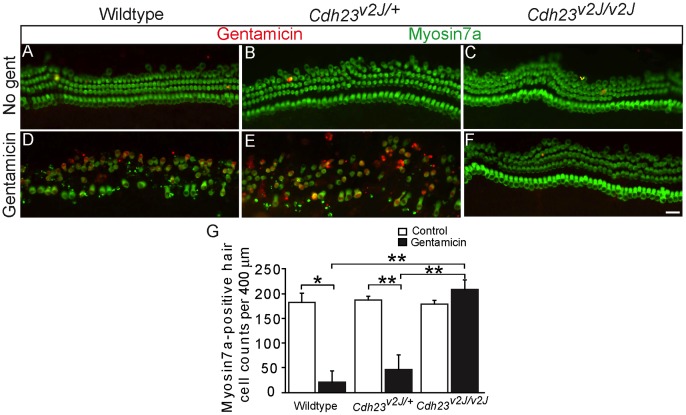
Cadherin 23 deficiency protects hair cells from gentamicin toxicity. P3 cochleae of mouse litters from *Cdh23^v2J/+^* breeding were cultured in control (A–C) or gentamicin-containing (0.25 mM) media (D–F). Cultured tissues were immunolabeled for myosin7a (green) and gentamicin (red). A–C) Untreated, cultured cochleae from wildtype, *Cdh23^v2J^* ^/+^, and *Cdh23^v2J/v2J^* mice exhibited an organized array of hair cells and no gentamicin labeling. D–E) Wildtype and *Cdh23^v2J^* ^/+^ littermates showed extensive hair cell loss and robust anti-gentamicin labeling following gentamicin treatment. F) *Cdh23^v2J/v2J^* cochleae exposed to gentamicin showed no hair cell loss or gentamicin labeling. G) Quantitative analyses show that hair cells from *Cdh23^v2J/v2J^* homozygous mice were significantly protected from gentamicin. * = p<0.01, ** = p<0.001, error bars = S.D., scale bar = 25 µm.

Because AG entry is necessary for hair cell toxicity [6], [38], we investigated whether a reduction of AG uptake would prevent loss of *Cdh23^v2J/v2J^* hair cells. Cochleae from *Cdh23^v2J^* homozygous and heterozygous mice were treated with gentamicin and hair cell survival was assessed and compared to those from wildtype littermates. Hair cell counts from untreated, cultured cochleae from the three genotypes were comparable: 177±33 (n = 3), 188±7 (n = 5), and 179±7 (n = 3) hair cells per 400 µm cochlear length in the middle turn of wildtype, *Cdh23^v2J/+^*, and *Cdh23^v2J/v2J^* cochleae, respectively (Figure 3A–C). Exposure to gentamicin (0.25 mM×1 hr) led to significant hair cell loss in the wildtype (34±45 hair cells, n = 9, p<0.01) and *Cdh23^v2J/+^* (47±30 hair cells, n = 19, p<0.001) cochleae two days later (Figure 3D–E, G), whereas Cdh23-deficient hair cells were protected from degeneration induced by gentamicin (208±19 hair cells, n = 6)(Figure 3F–G). In comparison to gentamicin-treated wildtype and *Cdh23^v2J/+^* cochleae, gentamicin-treated *Cdh23^v2J/c2J v2J^* cochleae exhibited significantly higher hair cell survival (p<0.001 for both). Immunofluorescent labeling for gentamicin detected drug uptake into wildtype and *Cdh23^v2J/+^* hair cells, but labeling was notably absent in *Cdh23^v2J/v2J^* hair cells. Together these data suggest that Cdh23 is required for AG uptake and its subsequent toxicity in sensory hair cells.

### Calcium Chelation Prevents AG Uptake and Hair Cell Loss

**Figure 4:**
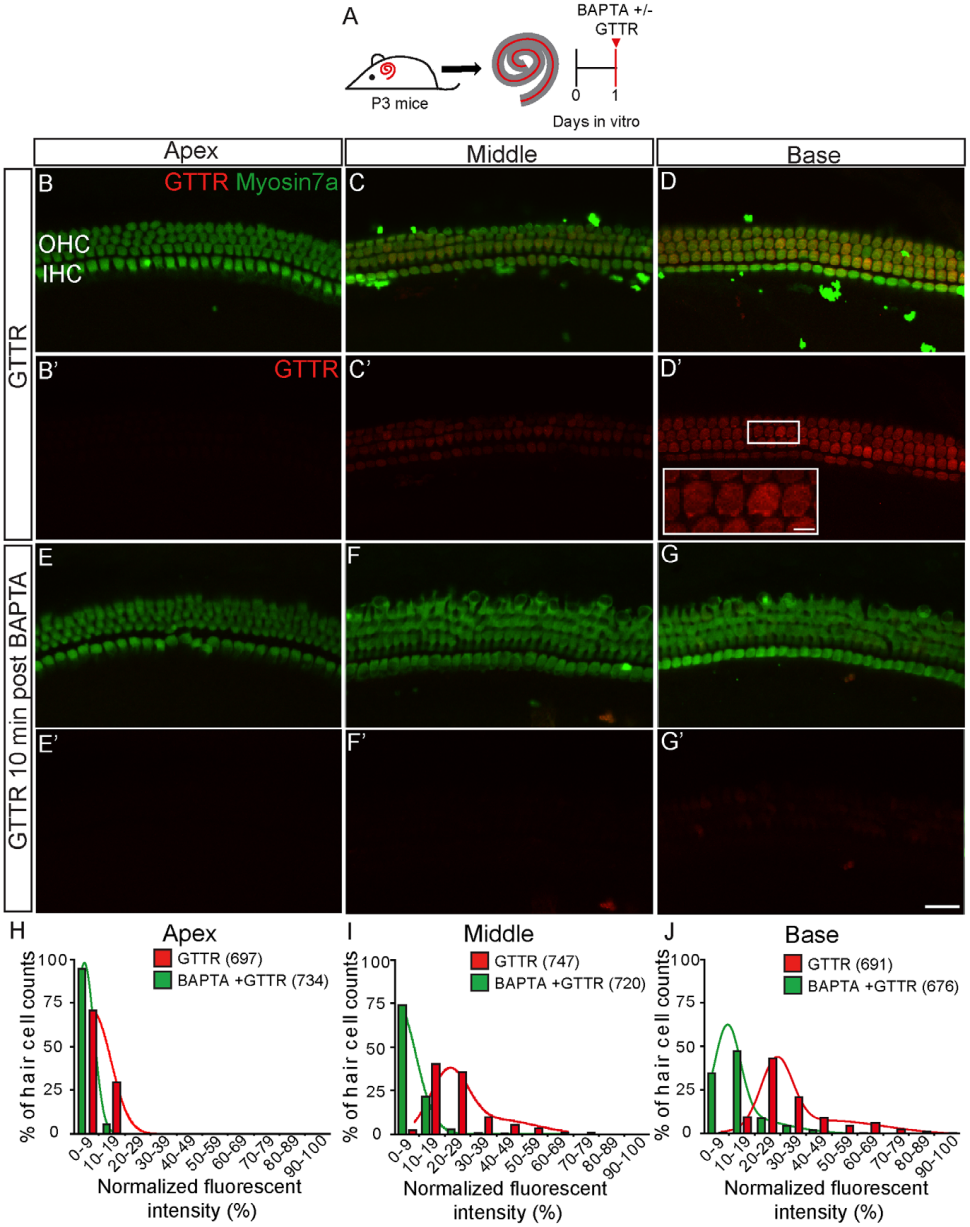
Disruption of tip-links with BAPTA diminishes GTTR uptake. A) P3 wildtype cochleae were cultured overnight, treated with BAPTA (5 mM), then exposed to GTTR (1 µM×1 hr). Control cultures were rinsed with BAPTA-free media and exposed to GTTR. All images were captured using identical microscope settings. B–D) In control cochleae, GTTR uptake into hair cells followed a basal-apical gradient, where hair cells in the basal turn were the most robustly labeled. E-G) BAPTA pre-treatment reduced GTTR uptake into hair cells throughout the cochlea. H-J) The fluorescence intensity of outer hair cells was quantified and normalized to the most intensely labeled cell among cultured organs at this time point. Histogram plots showing the distribution of normalized GTTR fluorescence intensity indicate that BAPTA exposure significantly reduced GTTR uptake (also see Table 1). A bimodal distribution was observed in the middle and basal turns. Scale bar = 25 µm in B–G and 5 µm in inset in D’.

Prior work suggested that Cdh23 mediates stereocilia development and so might confound the interpretation of the uptake experiments in some manner not directly related to MET channel gating [21], [37], [39]. To determine if an acute disruption of tip-link proteins would also affect AG entry, we treated hair cells with the calcium chelator 1,2-bis(o-aminophenoxy) ethane-N,N,N',N'-tetraacetic acid (BAPTA). The tip-link structures are calcium dependent and calcium chelation of hair cells leads to tip-link loss [16], [23]. Without prior calcium chelation, GTTR entered and labeled hair cells in a basal-apical gradient, where the most robustly labeled cells were found in the basal turn of the cochlea (Figure 4B–D). We quantified this basal-apical gradient of GTTR uptake by normalizing GTTR fluorescence intensities to the most intensely labeled hair cell, typically a basal cell (Table 1). Histogram plots show a best-fit Gaussian distribution of GTTR fluorescence intensities among apical hair cells (peak and full width half maximum (FWHM) = 5.7 and 16.5)(697 cells from 4 cochleae) and a bimodal distribution among hair cells from the middle (peaks and FWHM = 19.2 and 18.2 for the first peak and 40.0 and 35.3 for the second peak) and basal turns (peaks and FWHM = 26.3 and 14.4 for the first peak and 45.6 and 52.3 for the second peak)(747 and 691 cells from 4 cochleae, respectively)(Figure 4H–J, Table 1). In tissues pre-treated with BAPTA, GTTR uptake was remarkably reduced in hair cells of the middle and basal turns (Figure 4E–G). Quantification of GTTR fluorescence intensities in myosin7a-positive hair cells showed that BAPTA treatment reduced GTTR uptake by 82.3% (1 peak of 720 cells from 4 cochleae) in the middle turn and 58.2 and 75.9% (2 peaks of 676 cells from 4 cochleae) in basal turn (Figure 4H–J). The uptake of FM1-43 dye, a fluorescent dye known to permeate hair cell MET channels [5], [34], was similarly reduced in BAPTA-treated cochleae (Figure S2A–B).

**Table 1:** Gentamicin-conjugated Texas Red uptake in hair cells after calcium chelation with BAPTA++.

	Apex		Middle		Base	
Time after BAPTA	Control	BAPTA	Control	BAPTA	Control	BAPTA
10 min: Peak(s)(FWHM)*	5.7(16.5)	6.1(8.7)	19.2(18.2), 40.0(35.3)	3.4(17.3),	26.3(14.4), 45.6(52.3)	11.0(11.6),11.0(44.0),
6 hr: Peak(s)(FWHM)	7.0(9.2)	6.5(14.0),	12.2(8.6), 12.2(30.8)	7.7(14.7)	14.3(22.7), 59.6(50.8)	4.0(23.8),35.0(30.2)
24 hr: Peak(s)(FWHM)	15.3(11.5), 15.3(45.5)	13.9(12.3),	27.3(11.4), 41.8(35.8)	39.4(33.9),	52.7(43.6), 127.3(35.3)	49.5(35.5)

+ Normalized fluorescence intensity in outer hair cells from 4–6 cochleae.

* Full width half maximum.

**Figure 5:**
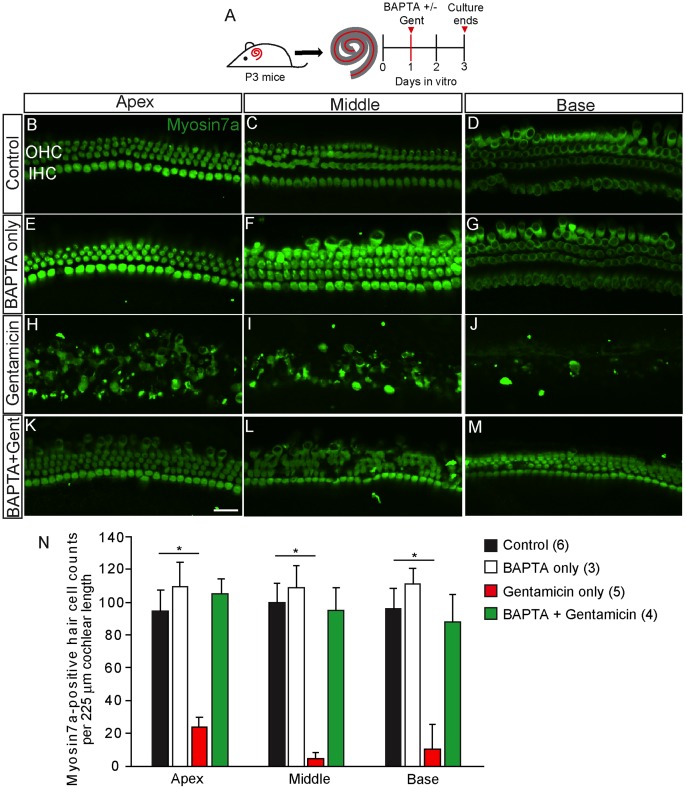
BAPTA pre-treatment reduces gentamicin toxicity in hair cells. A) Cultured cochleae from P3 wildtype mice were treated with BAPTA (5 mM) before gentamicin exposure (0.5 mM×1 hr). After an additional 48 hr AG-free recovery period, tissues were fixed and immunolabeled for myosin7a (green). B–G) Untreated cochlear cultures and cochlear organs treated with BAPTA only did not show hair cell loss. H–J) Gentamicin-treated cultures showed degeneration and a disarrayed arrangement of hair cells. K–M) Pre-treatment with BAPTA led to improved hair cell survival in comparison to treatment with gentamicin alone. N) Quantitative analysis of myosin7a-positive hair cell counts show significantly more hair cells in BAPTA pre-treated organs than those exposed to gentamicin alone. Error bars = S.D., * = p<0.001, scale bar = 25 µm.

We further tested whether calcium chelation conferred protection against AG toxicity by culturing cochleae with BAPTA and then gentamicin (0.5 mM×1hr), followed by a 48-hr AG-free, normocalcemic recovery period. When administered alone, gentamicin caused significant hair cell loss in the middle and basal turns (Figure 5H–J, N). BAPTA treatment, which by itself did not cause hair cell loss (Figure 5E–G, N), effectively prevented hair cell degeneration following AG treatment (Figure 5K–N). Immunofluorescent labeling for gentamicin was less prominent in cochleae exposed to both BAPTA and gentamicin compared to gentamicin alone (not shown). These data indicate that acute dissociation of tip-links via calcium chelation reduced AG uptake and toxicity in hair cells.

### Aminoglycosides Enter and Damage Hair Cells Recovering from Calcium Chelation

**Figure 6:**
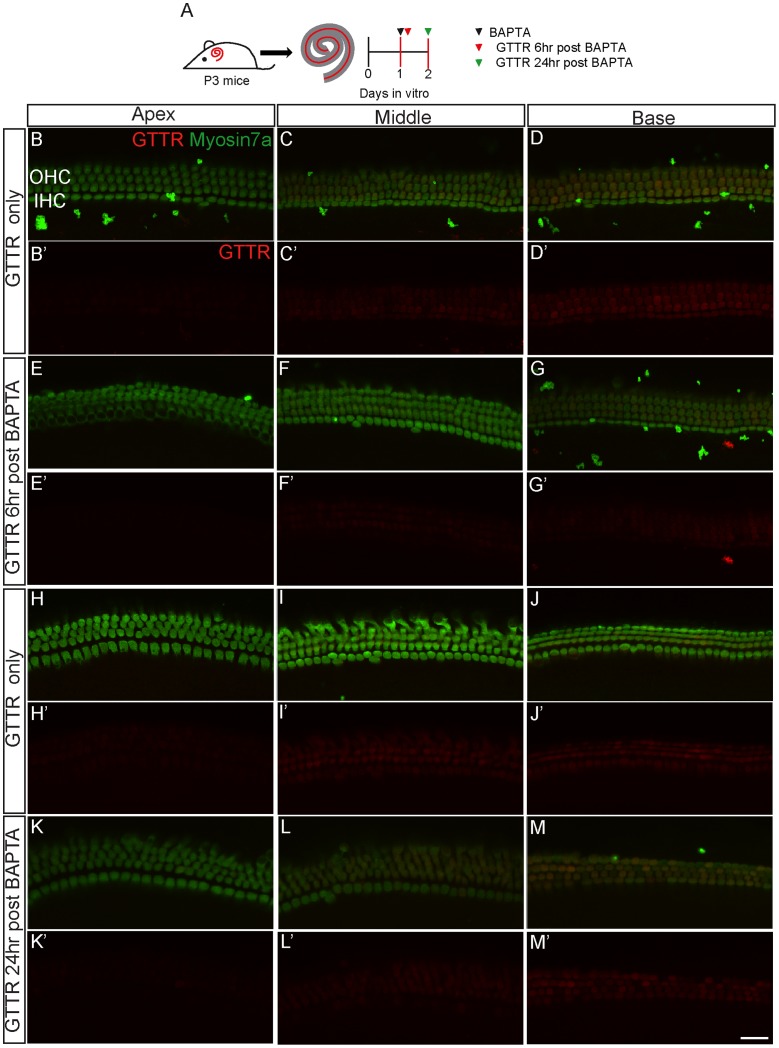
BAPTA-treated hair cells gradually regain ability to take up GTTR. A) Cochleae were cultured overnight, treated with BAPTA (5 mM), incubated in normocalcemic media for another 6 or 24 hr, and then exposed to GTTR (1 µM×1 hr). Control cochleae did not receive BAPTA treatment and were cultured for the same durations. B–D) At the 6 hr recovery time point, GTTR uptake was noted among hair cells from control tissues and followed a basal-apical gradient. E–G) GTTR uptake was diminished among BAPTA-treated organs. H–M) After another 24 hr in culture, BAPTA-treated hair cells and those from untreated, time-matched controls shared a similar degree and pattern of GTTR uptake. Scale bar = 25 µm.

**Figure 7:**
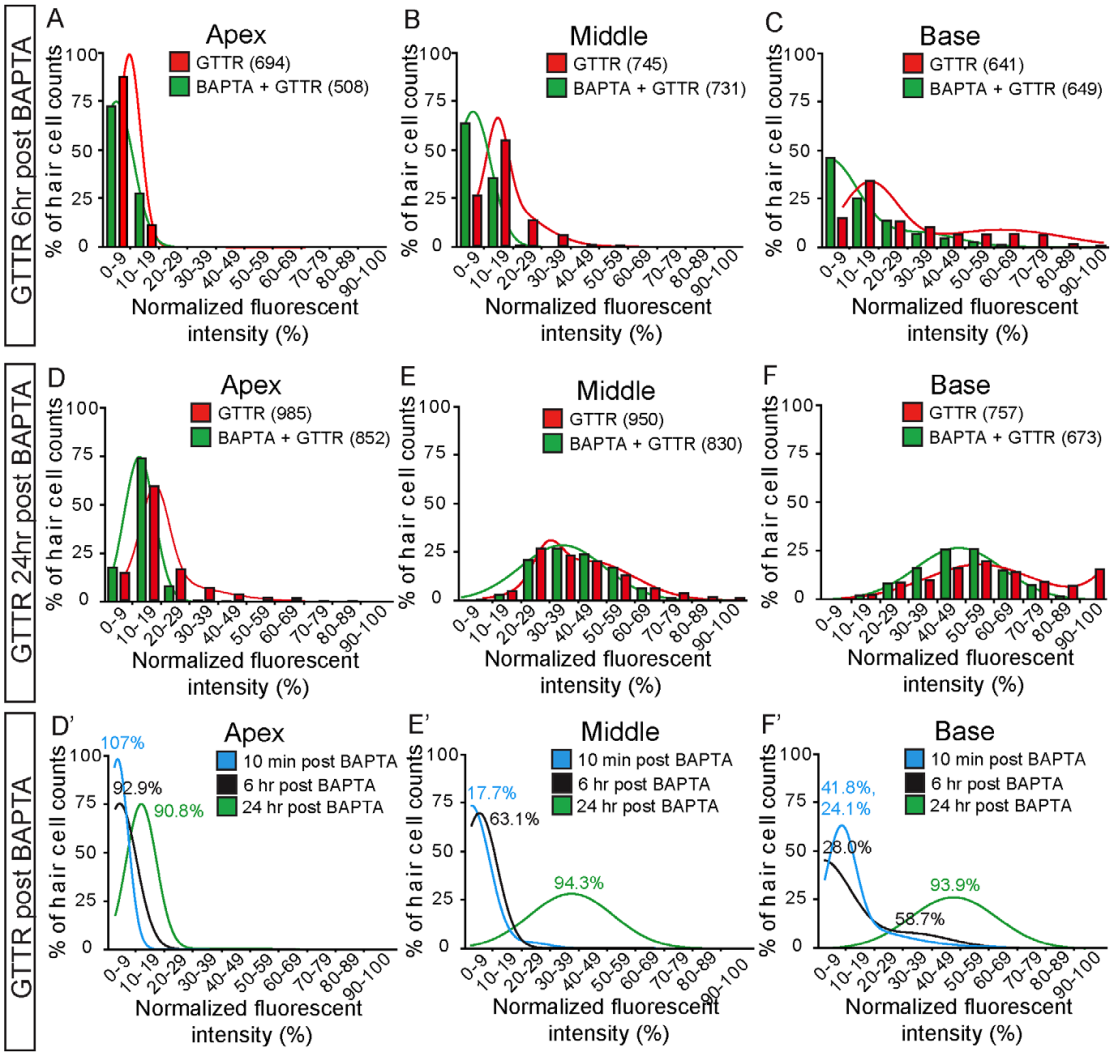
Histogram plots of hair cell GTTR fluorescence. A–C) Six hours after BAPTA treatment, hair cells showed diminished GTTR uptake (green) in comparison to untreated controls. Bimodal distributions were observed in both BAPTA-treated and untreated organs. D–F) Twenty-four hours after BAPTA exposure, GTTR uptake in treated hair cells (green) was comparable to controls (red). D’–F’) GTTR uptake gradually increased with longer durations after calcium chelation treatment. Percentages indicate fluorescence intensities from the BAPTA-treated group in comparison to those from the same cochlear turn from untreated, time-matched controls. When one peak is present in the BAPTA group and two peaks in control, the peak with a larger area under the curve was chosen for comparison.

The tip-link complex regenerates under normocalcemic conditions within 24 hours after disruption by calcium chelators [23], [24], [25]. To determine whether the mechanisms mediating AG uptake in hair cells recover from calcium chelation, we exposed cochleae to GTTR at 6 or 24 hours after BAPTA treatment. At 6 hours after BAPTA treatment, GTTR uptake was detectable among hair cells in the middle and basal turns of the cochlea, decreasing in a basal-apical gradient. As a group, BAPTA-treated cochleae showed significantly lower fluorescence intensities than those from untreated, time-matched controls (Figures 6B–G, 7A–C, Table 1). When compared to the same turns from untreated, time-matched controls, BAPTA-treated organs demonstrated lower GTTR fluorescence intensities in the middle (36.9%, single peak of 731 cells from 4 cochleae) and basal turns (41.3 and 72.0%, 2 peaks of 649 cells from 4 cochlea) (Figure 7D’–F’).

When GTTR was added 24 hours after BAPTA treatment, robust drug uptake was noted and the basal-apical gradient was maintained (Figure 6H–M). Quantification of fluorescence intensities showed that GTTR uptake was comparable between BAPTA-treated hair cells and those in parallel controls (Figure 7D–F, Table 1). In comparison to fluorescence intensity signals in the same turn in untreated, time-matched tissues, hair cells from middle and basal turns of BAPTA-treated cochleae have regained most of their ability to take up GTTR: 5.7% lower in the middle turn (830 cells from 6 cochleae) and 6.1% in the base (673 cells from 6 cochleae)(Figure 7D’–F’).

**Figure 8:**
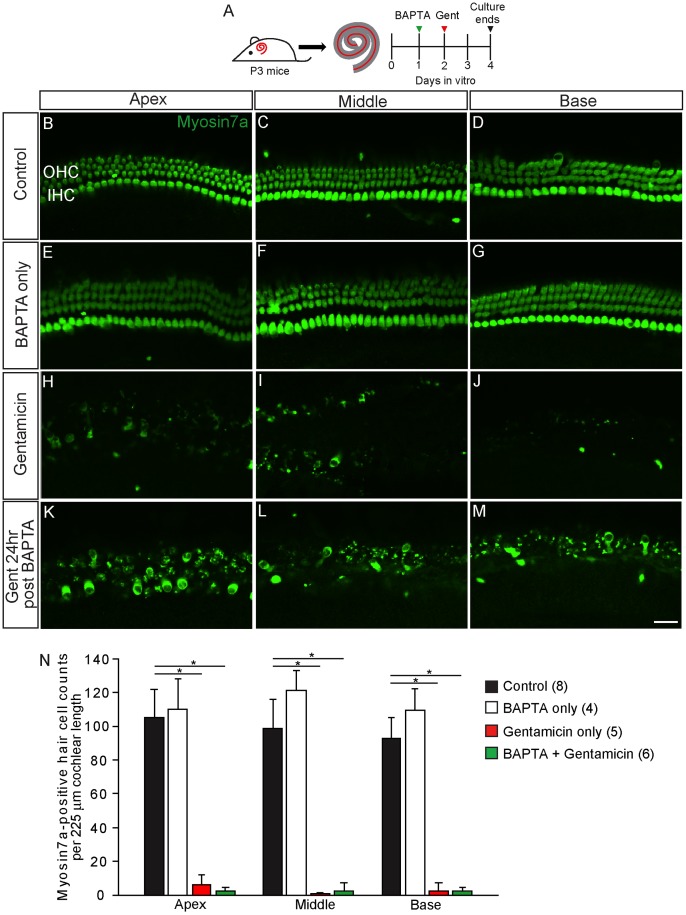
Hair cells recovered from BAPTA treatment were susceptible to damage by gentamicin. A) After BAPTA treatment, cochleae were incubated in BAPTA-free, normocalcemic media for 24 hr before exposure to gentamicin (0.5 mM×1 hr). Tissues were cultured for another 48 hr and then immunolabeled for myosin7a (green). B–G) Untreated cochlear cultures and cultures treated with BAPTA only did not show hair cell loss. H–M) Both cultures exposed to gentamicin alone or to gentamicin 24 hr after pre-treatment with BAPTA had extensive hair cell loss and disorganization. N) Quantitative analyses show that treatment with gentamicin alone or with gentamicin 24 hr after pre-treatment with BAPTA caused significant hair cell loss. Error bars = S.D., * = p<0.001, scale bar = 25 µm.

To determine if this recovered ability for AG to enter hair cells can lead to toxicity, we applied gentamicin (0.5 mM×1 hr) to cochleae 24 hours after BAPTA treatment and allowed an additional 48-hour AG-free culture period (Figure 8A). With this treatment paradigm, we found significant hair cell loss in organs pre-treated with BAPTA followed by gentamicin to an extent comparable to gentamicin treatment alone (Figure 8B–NO). These lines of evidence suggest that sensory hair cells exposed to calcium chelation regenerate the machinery necessary for AG uptake and toxicity.

## Discussion

Inner ear sensory hair cells are essential for normal auditory function. Preventable insults including noise and AGs can cause hair cell degeneration and permanent hearing loss [2], [3]. Because drug entry is necessary for toxicity, understanding AG entry has important therapeutic implications. Here, we show that the tip-link complex is required for AG entry and toxicity in cochlear hair cells.

Independent lines of research have demonstrated that AGs enter hair cells via the MET channels and that blocking this entry route prevented toxicity [4], [5], [6]. Alternative uptake pathways such as via endocytosis and transient receptor potential channels have also been described in sensory hair cells [13], [14], [38]. Upon calcium chelation, tip-link structures and MET channel currents are lost, both of which spontaneously return within 24 hours [23], [24], [25]. Our observation that AG entry and toxicity were temporarily suppressed after BAPTA treatment correlated with the reported time course of initial decrease and subsequent return of MET channel currents [24], [25], thus supporting the model where AGs enter hair cells via the MET channels and that patent MET channels are required for AG toxicity (Figure 1C) [6].

Alagramam et al. examined mouse models deficient in the tip-link protein Pcdh15, the proposed binding partner of Cdh23 and found reduced uptake of gentamicin in *Pcdh15^av6J/av6J^* mutants and no uptake in *Pcdh15^av3J/av3J^* mutants [17]. However, *Pcdh15^av6J/av6J^* hair cells were susceptible to hair cell degeneration caused by gentamicin, while *Pcdh15^av3J/av3J^* hair cells were protected. Interestingly, this difference in AG uptake reduction correlated with the degree of morphological integrity of tip-link-like structures between the two mouse models [17]. In support of the notion that tip-link integrity predicts AG uptake and toxicity in hair cells, *Cdh23^v2J/v2J^* hair cells, like *Pcdh15^av3J/av3J^* hair cells, are deficient in tip-links under scanning electron microscopy [17] and are shown in the current study to fail to take up AG and thus protected from its damage.

As a component of the tip-link complex in mature hair cells, Cdh23 has also been implicated in stereocilia development [21], [37], [39], [40]. While one interpretation of the decreased AG uptake and toxicity in *Cdh23^v2J/v2J^* hair cells is that they resulted from dysfunctional tip-links, it is possible that other members of the MET machinery, some of which have yet to be identified, were misregulated during development as a result of Cdh23 deficiency. In support of this possibility, electrophysiological measurements of *Cdh23^v2J/v2J^* hair cells found both reduced mechanosensitivity and abnormal directional sensitivity [17]. Nonetheless, Cdh23 is necessary for tip-link development and regeneration after calcium chelation as protein fragments masking the putative binding domains of Cdh23 effectively prevented these events [24].

One proposed explanation for the basal-apical gradient of AG toxicity is that basal hair cells contain larger MET currents facilitating AG entry [6], [35]. In support of this hypothesis, the patterns of GTTR uptake and hair cell loss both follow such a gradient. Upon regeneration of tip-links, AG uptake and toxicity return to hair cells and their extents again follow a basal-apical gradient, suggesting that while the tip-link complex is required for the re-establishment of MET currents along the cochlear turns, it likely does not play a role in maintaining the tonotopic gradient of MET current amplitudes, which remained preserved after regeneration of tip-links. The precise mechanisms controlling the cochlear tonotopy are yet to be determined [41].

In the neonatal cochlea, maturing hair cells acquire a step-wise increase in MET channel currents [6], [10]. This developmental process likely mediates the increased AG uptake and toxicity observed in more mature hair cells, a relationship also noted in the zebrafish lateral line system [9]. Upon calcium chelation, AG uptake and toxicity were temporarily suppressed and subsequently returned in hair cells to an extent comparable to untreated, time-matched cochleae (Figure 6H–M, 7D–F). Therefore, a temporary break in tip-links is unlikely to impact the maturation process of MET channel amplitude that normally occurs *in vitro* [10].

In birds, acoustic trauma induces loss of tip-links followed by a partial return [42], [43]. Although the time course of regeneration *in vitro* closely parallels that of temporary threshold shifts, whether tip-links similarly break and recover in mammals after noise is unclear. It is long known that antecedent noise exposure exacerbates the toxicity resulting from subsequent aminoglycoside administration [44]. Recently, Li et al. demonstrated that prior temporary threshold shifts caused by chronic noise exposure led to enhanced AG accumulation in sensory hair cells [45]. This finding may result from increased drug trafficking across the blood-labyrinth barrier [45], thus raising AG concentration in the endolymph compartment where hair cell stereocilia reside. Indeed, increased entry of other cationic compounds comparable to AG have been observed after noise exposure [46]. However, it is important to note that the temporal relationship between noise and AG treatments influences their combined effects on the cochlea as concurrent exposure failed to potentiate AG uptake [45] and only induced a modest increase in hair cell loss and threshold shift [44]. In addition to the breakdown of blood-labyrinth barrier and hair cell tip-links, it is probable that other cellular changes occurring during temporary threshold shifts can influence AG uptake and/or toxicity in hair cells, further complicating the relationship between acoustic trauma and AG ototoxicity. It would be of interest to evaluate whether an acute and selective loss of tip-links causes temporary threshold shifts preventing aminoglycoside uptake and toxicity *in vivo*.

In summary, our data show that tip-links, as components of the mechanotransduction machinery, are required for AG uptake and its subsequent toxicity in sensory hair cells. Because tip-links readily regenerate, our study provides insights into a possible therapeutic target to limit AG entry and toxicity in sensory hair cells. However, such an approach will require better understanding of AG entry mechanisms and its subsequent effects on other cochlear cell types.

## Supporting Information

Figure S1 **Comparison of BAPTA application methods on gentamicin-induced hair cell toxicity.** The dripping method involves adding droplets of BAPTA-containing media directly on top of the cochlea, whereas BAPTA-containing media was added as a steady stream aimed directly at the cochlea in the squirting method. A) Both methods provided significantly improved hair cell survival, although we observed higher variability with the dripping method as indicated by larger standard deviations in all three turns. B) Twenty-four hours after BAPTA treatment, organs treated with either method showed comparable degrees of hair cell loss to organs treated with gentamicin alone. Error bars = S.D., * = p<0.01.
(TIFF)

Figure S2 **BAPTA treatment reduces FM1-43 dye uptake into hair cells.** Shown are representative images of middle turns of cochleae (n ≥3) treated with FM1-43 (5 µM×15 sec)(A) or pre-treated with BAPTA before FM1-43 administration (B). Live tissues were imaged using identical microscope settings. Robust labeling of hair cells by FM1-43 was notably reduced after BAPTA treatment. GER = greater epithelial ridge. Scale bar = 25 µm.
(TIFF)

## References

[1] Rizzi MD, Hirose K (2007) Aminoglycoside ototoxicity. Curr Opin Otolaryngol Head Neck Surg 15: 352–357. 17823553 10.1097/MOO.0b013e3282ef772d

[2] Forge A, Schacht J (2000) Aminoglycoside antibiotics. Audiol Neurootol 5: 3–22. 10686428 10.1159/000013861

[3] Huth ME, Ricci AJ, Cheng AG (2011) Mechanisms of aminoglycoside ototoxicity and targets of hair cell protection. Int J Otolaryngol 2011: 937861. 22121370 10.1155/2011/937861 PMC3202092

[4] Marcotti W, van Netten SM, Kros CJ (2005) The aminoglycoside antibiotic dihydrostreptomycin rapidly enters mouse outer hair cells through the mechano-electrical transducer channels. J Physiol 567: 505–521. 15994187 10.1113/jphysiol.2005.085951 PMC1474200

[5] Gale JE, Marcotti W, Kennedy HJ, Kros CJ, Richardson GP (2001) FM1–43 dye behaves as a permeant blocker of the hair-cell mechanotransducer channel. J Neurosci 21: 7013–7025. 11549711 10.1523/JNEUROSCI.21-18-07013.2001 PMC6762973

[6] Alharazneh A, Luk L, Huth M, Monfared A, Steyger PS, et al (2011) Functional hair cell mechanotransducer channels are required for aminoglycoside ototoxicity. PLoS One 6: e22347. 21818312 10.1371/journal.pone.0022347 PMC3144223

[7] Coffin AB, Reinhart KE, Owens KN, Raible DW, Rubel EW (2009) Extracellular divalent cations modulate aminoglycoside-induced hair cell death in the zebrafish lateral line. Hear Res 253: 42–51. 19285547 10.1016/j.heares.2009.03.004 PMC5139914

[8] Ricci A (2002) Differences in mechano-transducer channel kinetics underlie tonotopic distribution of fast adaptation in auditory hair cells. J Neurophysiol 87: 1738–1748. 11929895 10.1152/jn.00574.2001

[9] Santos F, MacDonald G, Rubel EW, Raible DW (2006) Lateral line hair cell maturation is a determinant of aminoglycoside susceptibility in zebrafish (Danio rerio). Hear Res 213: 25–33. 16459035 10.1016/j.heares.2005.12.009

[10] Waguespack J, Salles FT, Kachar B, Ricci AJ (2007) Stepwise morphological and functional maturation of mechanotransduction in rat outer hair cells. J Neurosci 27: 13890–13902. 18077701 10.1523/JNEUROSCI.2159-07.2007 PMC6673611

[11] Richardson GP, Forge A, Kros CJ, Fleming J, Brown SD, et al (1997) Myosin VIIA is required for aminoglycoside accumulation in cochlear hair cells. J Neurosci 17: 9506–9519. 9391006 10.1523/JNEUROSCI.17-24-09506.1997 PMC6573420

[12] Kawashima Y, Geleoc GS, Kurima K, Labay V, Lelli A, et al (2011) Mechanotransduction in mouse inner ear hair cells requires transmembrane channel-like genes. J Clin Invest 121: 4796–4809. 22105175 10.1172/JCI60405 PMC3223072

[13] Hashino E, Shero M (1995) Endocytosis of aminoglycoside antibiotics in sensory hair cells. Brain Res 704: 135–140. 8750975 10.1016/0006-8993(95)01198-6

[14] Stepanyan RS, Indzhykulian AA, Velez-Ortega AC, Boger ET, Steyger PS, et al (2011) TRPA1-mediated accumulation of aminoglycosides in mouse cochlear outer hair cells. J Assoc Res Otolaryngol 12: 729–740. 21879401 10.1007/s10162-011-0288-x PMC3214240

[15] Osborne MP, Comis SD, Pickles JO (1984) Morphology and cross-linkage of stereocilia in the guinea-pig labyrinth examined without the use of osmium as a fixative. Cell Tissue Res 237: 43–48. 6332677 10.1007/BF00229198

[16] Assad JA, Shepherd GM, Corey DP (1991) Tip-link integrity and mechanical transduction in vertebrate hair cells. Neuron 7: 985–994. 1764247 10.1016/0896-6273(91)90343-x

[17] Alagramam KN, Goodyear RJ, Geng R, Furness DN, van Aken AF, et al (2011) Mutations in protocadherin 15 and cadherin 23 affect tip links and mechanotransduction in mammalian sensory hair cells. PLoS One 6: e19183. 21532990 10.1371/journal.pone.0019183 PMC3080917

[18] Kazmierczak P, Sakaguchi H, Tokita J, Wilson-Kubalek EM, Milligan RA, et al (2007) Cadherin 23 and protocadherin 15 interact to form tip-link filaments in sensory hair cells. Nature 449: 87–91. 17805295 10.1038/nature06091

[19] Rzadzinska AK, Derr A, Kachar B, Noben-Trauth K (2005) Sustained cadherin 23 expression in young and adult cochlea of normal and hearing-impaired mice. Hear Res 208: 114–121. 16005171 10.1016/j.heares.2005.05.008

[20] Sollner C, Rauch GJ, Siemens J, Geisler R, Schuster SC, et al (2004) Mutations in cadherin 23 affect tip links in zebrafish sensory hair cells. Nature 428: 955–959. 15057246 10.1038/nature02484

[21] Siemens J, Lillo C, Dumont RA, Reynolds A, Williams DS, et al (2004) Cadherin 23 is a component of the tip link in hair-cell stereocilia. Nature 428: 950–955. 15057245 10.1038/nature02483

[22] Ahmed ZM, Goodyear R, Riazuddin S, Lagziel A, Legan PK, et al (2006) The tip-link antigen, a protein associated with the transduction complex of sensory hair cells, is protocadherin-15. J Neurosci 26: 7022–7034. 16807332 10.1523/JNEUROSCI.1163-06.2006 PMC6673907

[23] Zhao Y, Yamoah EN, Gillespie PG (1996) Regeneration of broken tip links and restoration of mechanical transduction in hair cells. Proc Natl Acad Sci U S A 93: 15469–15474. 8986835 10.1073/pnas.93.26.15469 PMC26428

[24] Lelli A, Kazmierczak P, Kawashima Y, Muller U, Holt JR (2010) Development and regeneration of sensory transduction in auditory hair cells requires functional interaction between cadherin-23 and protocadherin-15. J Neurosci 30: 11259–11269. 20739546 10.1523/JNEUROSCI.1949-10.2010 PMC2949085

[25] Jia S, Yang S, Guo W, He DZ (2009) Fate of mammalian cochlear hair cells and stereocilia after loss of the stereocilia. J Neurosci 29: 15277–15285. 19955380 10.1523/JNEUROSCI.3231-09.2009 PMC2795320

[26] Marquis RE, Hudspeth AJ (1997) Effects of extracellular Ca2+ concentration on hair-bundle stiffness and gating-spring integrity in hair cells. Proc Natl Acad Sci U S A 94: 11923–11928. 9342338 10.1073/pnas.94.22.11923 PMC23657

[27] Crawford AC, Evans MG, Fettiplace R (1991) The actions of calcium on the mechano-electrical transducer current of turtle hair cells. J Physiol 434: 369–398. 1708822 10.1113/jphysiol.1991.sp018475 PMC1181423

[28] Rzadzinska AK, Steel KP (2009) Presence of interstereocilial links in waltzer mutants suggests Cdh23 is not essential for tip link formation. Neuroscience 158: 365–368. 18996172 10.1016/j.neuroscience.2008.10.012 PMC2989438

[29] Senften M, Schwander M, Kazmierczak P, Lillo C, Shin JB, et al (2006) Physical and functional interaction between protocadherin 15 and myosin VIIa in mechanosensory hair cells. J Neurosci 26: 2060–2071. 16481439 10.1523/JNEUROSCI.4251-05.2006 PMC2712835

[30] Chai R, Xia A, Wang T, Jan TA, Hayashi T, et al (2011) Dynamic expression of Lgr5, a Wnt target gene, in the developing and mature mouse cochlea. J Assoc Res Otolaryngol 12: 455–469. 21472479 10.1007/s10162-011-0267-2 PMC3123443

[31] Sobkowicz HM, Loftus JM, Slapnick SM (1993) Tissue culture of the organ of Corti. Acta Otolaryngol Suppl 502: 3–36. 8475741

[32] Richardson GP, Russell IJ (1991) Cochlear cultures as a model system for studying aminoglycoside induced ototoxicity. Hear Res 53: 293–311. 1880082 10.1016/0378-5955(91)90062-e

[33] Wang Q, Steyger PS (2009) Trafficking of systemic fluorescent gentamicin into the cochlea and hair cells. J Assoc Res Otolaryngol 10: 205–219. 19255807 10.1007/s10162-009-0160-4 PMC2674203

[34] Meyers JR, MacDonald RB, Duggan A, Lenzi D, Standaert DG, et al (2003) Lighting up the senses: FM1–43 loading of sensory cells through nonselective ion channels. J Neurosci 23: 4054–4065. 12764092 10.1523/JNEUROSCI.23-10-04054.2003 PMC6741082

[35] Ricci AJ, Crawford AC, Fettiplace R (2003) Tonotopic variation in the conductance of the hair cell mechanotransducer channel. Neuron 40: 983–990. 14659096 10.1016/s0896-6273(03)00721-9

[36] Tiede LM, Rocha-Sanchez SM, Hallworth R, Nichols MG, Beisel K (2007) Determination of hair cell metabolic state in isolated cochlear preparations by two-photon microscopy. J Biomed Opt 12: 021004. 17477711 10.1117/1.2714777 PMC1992521

[37] Di Palma F, Holme RH, Bryda EC, Belyantseva IA, Pellegrino R, et al (2001) Mutations in Cdh23, encoding a new type of cadherin, cause stereocilia disorganization in waltzer, the mouse model for Usher syndrome type 1D. Nat Genet 27: 103–107. 11138008 10.1038/83660

[38] Hiel H, Schamel A, Erre JP, Hayashida T, Dulon D, et al (1992) Cellular and subcellular localization of tritiated gentamicin in the guinea pig cochlea following combined treatment with ethacrynic acid. Hear Res 57: 157–165. 1733909 10.1016/0378-5955(92)90148-g

[39] Michel V, Goodyear RJ, Weil D, Marcotti W, Perfettini I, et al (2005) Cadherin 23 is a component of the transient lateral links in the developing hair bundles of cochlear sensory cells. Dev Biol 280: 281–294. 15882573 10.1016/j.ydbio.2005.01.014

[40] Lagziel A, Ahmed ZM, Schultz JM, Morell RJ, Belyantseva IA, et al (2005) Spatiotemporal pattern and isoforms of cadherin 23 in wild type and waltzer mice during inner ear hair cell development. Dev Biol 280: 295–306. 15882574 10.1016/j.ydbio.2005.01.015

[41] Mann ZF, Kelley MW (2011) Development of tonotopy in the auditory periphery. Hear Res 276: 2–15. 21276841 10.1016/j.heares.2011.01.011

[42] Kurian R, Krupp NL, Saunders JC (2003) Tip link loss and recovery on chick short hair cells following intense exposure to sound. Hear Res 181: 40–50. 12855361 10.1016/s0378-5955(03)00165-5

[43] Husbands JM, Steinberg SA, Kurian R, Saunders JC (1999) Tip-link integrity on chick tall hair cell stereocilia following intense sound exposure. Hear Res 135: 135–145. 10491962 10.1016/s0378-5955(99)00101-x

[44] Ryan AF, Bone RC (1982) Non-simultaneous Interaction of exposure to noise and kanamycin intoxication in the chinchilla. Am J Otolaryngol 3: 264–272. 7149139 10.1016/s0196-0709(82)80065-3

[45] Li H, Wang Q, Steyger PS (2011) Acoustic trauma increases cochlear and hair cell uptake of gentamicin. PLoS One 6: e19130. 21552569 10.1371/journal.pone.0019130 PMC3084257

[46] Suzuki M, Yamasoba T, Ishibashi T, Miller JM, Kaga K (2002) Effect of noise exposure on blood-labyrinth barrier in guinea pigs. Hear Res 164: 12–18. 11950520 10.1016/s0378-5955(01)00397-5

