# Supplementary figures and images for: Integrity and Regeneration of Mechanotransduction Machinery Regulate Aminoglycoside Entry and Sensory Cell Death
Source: PLoS One. 2013 Jan 24;8(1):e54794. doi: 10.1371/journal.pone.0054794 (PMC3554584; doi:10.1371/journal.pone.0054794)

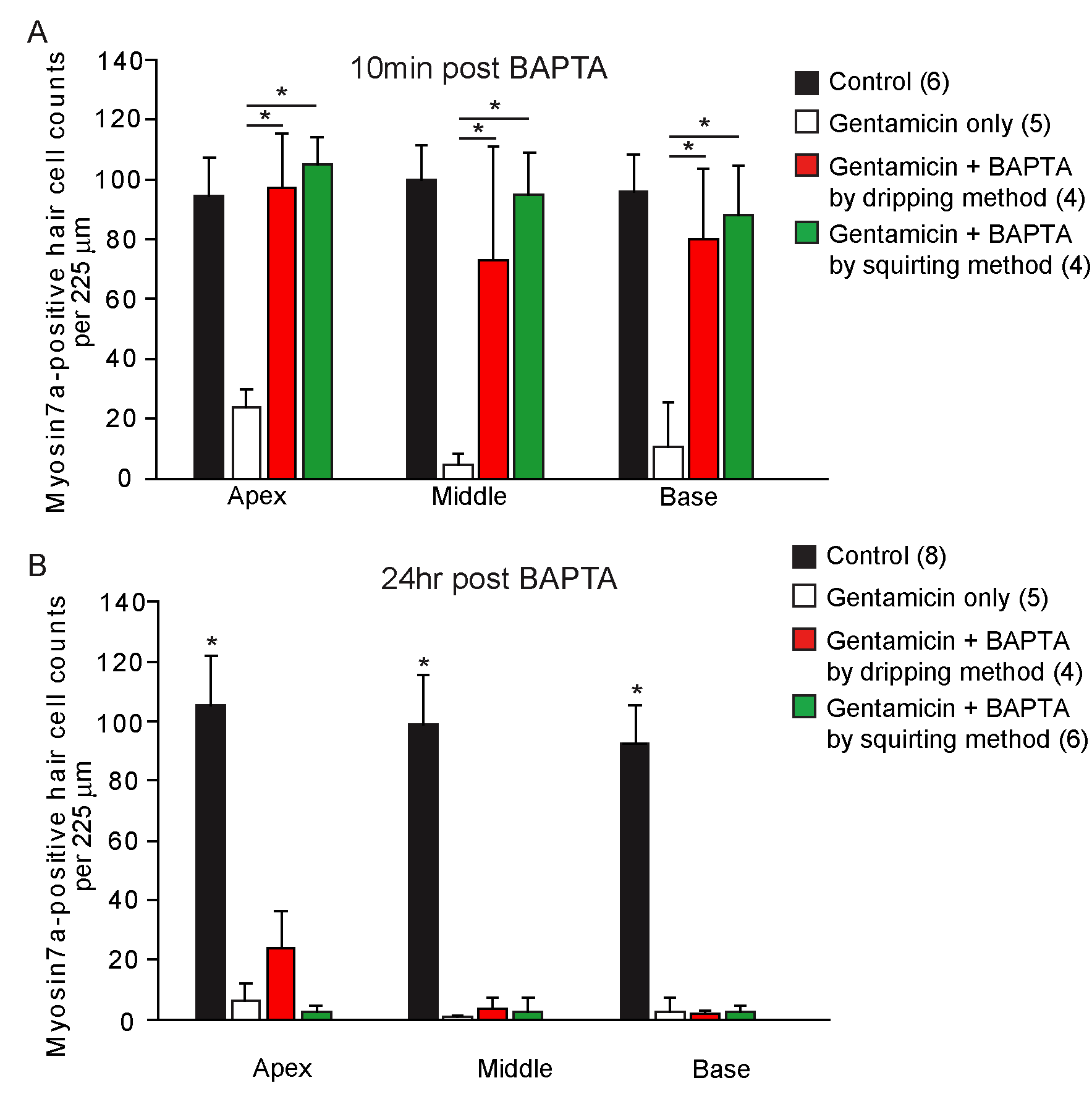

Supplement: Figure S1 — Comparison of BAPTA application methods on gentamicin-induced hair cell toxicity. The dripping method involves adding droplets of BAPTA-containing media directly on top of the cochlea, whereas BAPTA-containing media was added as a steady stream aimed directly at the cochlea in the squirting method. A) Both methods provided significantly improved hair cell survival, although we observed higher variability with the dripping method as indicated by larger standard deviations in all three turns. B) Twenty-four hours after BAPTA treatment, organs treated with either method showed comparable degrees of hair cell loss to organs treated with gentamicin alone. Error bars = S.D., * = p<0.01. (TIFF) [file pone.0054794.s001.tiff]

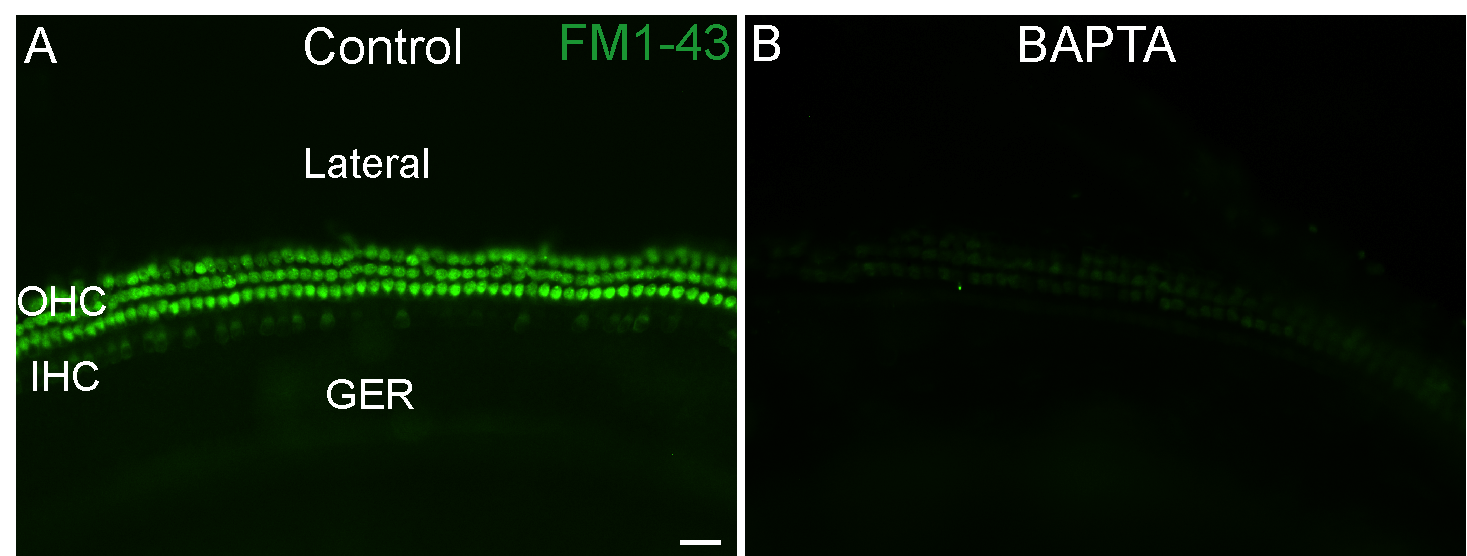

Supplement: Figure S2 — BAPTA treatment reduces FM1-43 dye uptake into hair cells. Shown are representative images of middle turns of cochleae (n ≥3) treated with FM1-43 (5 µM×15 sec)(A) or pre-treated with BAPTA before FM1-43 administration (B). Live tissues were imaged using identical microscope settings. Robust labeling of hair cells by FM1-43 was notably reduced after BAPTA treatment. GER = greater epithelial ridge. Scale bar = 25 µm. (TIFF) [file pone.0054794.s002.tiff]
